# Case Report: effect of lumasiran treatment in a late preterm baby with antenatal diagnosis of primary hyperoxaluria type 1

**DOI:** 10.3389/fped.2023.1338909

**Published:** 2024-01-16

**Authors:** Francesca Taroni, Alfredo Berrettini, Michele Gnech, Francesca Rella, Gian Antonio Manzoni, Giovanni Montini

**Affiliations:** ^1^ Pediatric Nephrology, Dialysis and Transplant Unit, Fondazione IRCCS Ca’ Granda-Ospedale Maggiore Policlinico, Milano, Italy; ^2^ Department of Pediatric Urology, Fondazione IRCCS Ca’ Granda Ospedale Maggiore Policlinico, Milano, Italy; ^3^ Department of Clinical Sciences and Community Health, University of Milan, Milan, Italy

**Keywords:** late preterm, lumasiran, newborn, prenatal diagnosis, primary hyperoxaluria type 1

## Abstract

**Background:**

Primary hyperoxaluria type 1 (PH1) is a rare disease with autosomal recessive transmission, characterized by increased urinary excretion of oxalate, resulting in chronic kidney disease secondary to recurrent urolithiasis, nephrocalcinosis, and accumulation of oxalate in various organs and tissues (systemic oxalosis). Since 2020, an innovative pharmacological approach, namely, lumasiran, has been added to the therapeutic armamentarium (dialysis and liver-kidney transplantation). The purpose of this paper is to describe the effect of lumasiran initiated at 10 days of life in a newborn with prenatally diagnosed PH1. A female fetus was prenatally diagnosed with hyperoxaluria type 1, based on family history and genetic testing. Her brother had the onset of the disease at 2 months of age and underwent liver and kidney transplantation at 13 months and 8 years of age, respectively. The baby was born late preterm at 36 weeks + 4 days of gestation via spontaneous labor, and lumasiran for compassionate use was started on the tenth day of life. At 20 months of age, the baby showed normal urinary oxalate values and kidney function, while the plasma oxalate level was under the threshold of oversaturation. There were no signs of systemic oxalosis.

**Conclusions:**

Early use of lumasiran in young infants, who do not yet show signs of the disease, represents a therapeutic challenge for the pediatric nephrologist. The ability of the drug to act on the hepatocyte of the newborn and the most appropriate dosage to be used in these very young babies have yet to be clarified.

## Introduction

Primary hyperoxaluria type 1 (PH1) is a rare autosomal recessive inherited disease characterized by increased supersaturation of oxalate, resulting in chronic kidney disease and the need for replacement therapy, secondary to recurrent urolithiasis and nephrocalcinosis, and accumulation of oxalate in various organs and tissues (systemic oxalosis) (1). The median age at onset of the disease is 5.5 years, but the disease can be diagnosed at any age, from birth to the sixth decade of life (2).

PH1 is caused by mutations in the AGXT gene, which codes for the liver enzyme L-alanine glyoxylate aminotransferase (AGT). When AGT activity is absent or reduced, there is an accumulation of glyoxylate that is converted by hepatic lactate dehydrogenase (LDH) to oxalate, the overproduction of which results in hyperoxaluria and hyperoxalemia (3–8).

Until 2020, the mainstays of therapy for PH1 were conservative therapy, dialysis, and liver-kidney transplantation. Currently, an innovative pharmacological approach based on the use of siRNA, such as lumasiran and nedosiran, has been added to the therapeutic armamentarium. Nedosiran inhibits the production of L-lactate dehydrogenase A (LDHA), which is essential for the cytosolic conversion of glyoxylate into oxalate. Lumasiran is designed to silence the gene that encodes the enzyme glycolate oxidase, which catalyzes the conversion of glycolate into glyoxylate (9). The ILLUMINATE A, B, and C studies have provided interesting evidence regarding the efficacy and safety of lumasiran. Data relating to the efficacy of lumasiran in the pediatric cohort are supported by the ILLUMINATE B trial in which 18 patients aged between 3 months and 6 years were enrolled. Therefore, experience of the use of lumasiran in newborns is limited to date (10–12).

The purpose of this paper is to describe the effect of lumasiran initiated very early in life in a newborn with prenatally diagnosed PH1.

## Case description

A female fetus was prenatally diagnosed with hyperoxaluria type 1 based on family history and genetic testing.

Her brother, currently 9 years old, had the onset of disease at 2 months of age with a presentation of kidney damage and nephrocalcinosis, followed by genetic testing for PH1, which showed a compound heterozygous mutation of the AGXT gene (c.466G>A-p. (Gly156Arg)-c.943-1G>T), which was resistant to pyridoxine. At 13 months of life, the proband's sibling had a CKD stage 3 estimated by the Schwartz formula, and isolated liver transplantation was performed. GFR showed a slow decrease in the following years, reaching a value of 15 ml/min/1.73 m^2^ at 7 years of age. Therefore, hemodialysis was started and the child underwent a kidney transplant from a living donor (his father) at the age of 8 years.

Given the family history, an indication was made to perform amniocentesis, which confirmed that the fetus had the same mutation as the brother. Fetal ultrasound scans and amniotic fluid were normal, with a regular course of pregnancy.

The girl was born via spontaneous labor at 36 weeks + 4 days of gestation with an adequate birth weight (2,615 grams). Hyperidratation by intravenous fluids (glucosaline solution, 70 ml/kg) and complementary breastfeeding were started, preventing weight loss of more than 5% of birth weight. Intravenous fluid administration was stopped on day 7 of life when the infant reached birth weight after a weight loss of 3% of birth weight. Then a fluid intake of 1.5 L/m^2^ was given to maintain high hydration by breastfeeding and complementary breastfeeding.

At 72 h of life, urinary oxalate levels were 318 mMol/Mol creatinine (normal range <300 mMol/Mol creatinine) with normal kidney function (creatinine 0.8 mg/dl). At 7 days of age, urinary oxalate levels were 1,200 mMol/Mol and the plasma oxalate level was 68 μMol/L, with a normal range being <4 μMol/L. Serum creatinine on day 7 was 0.68 mg/dl.

Lumasiran was initiated for compassionate use on the tenth day of life, according to the product's prescribing information. Lumasiran was administered according to the following schedule: 3 mg/kg once monthly until 10 kg and then at a dose of 6 mg/kg every 3 months. The baby also continued to receive hyperhydration with complementary breastfeeding, and from the first month of life alkalizing therapy with potassium citrate. Urinary oxalate concentrations started to reduce at day 21 (11 days after the first dose of lumasiran) and reached normal value at 9 months. Plasma oxalate levels were 10 uMol/L under the value of oversaturation at 6 months (20 uMol/L) (9).

At 7 days of age, the first ultrasound scan of the kidneys and urinary tract was performed, which showed well-differentiated kidneys with normal echogenicity.

**Figure 1:**
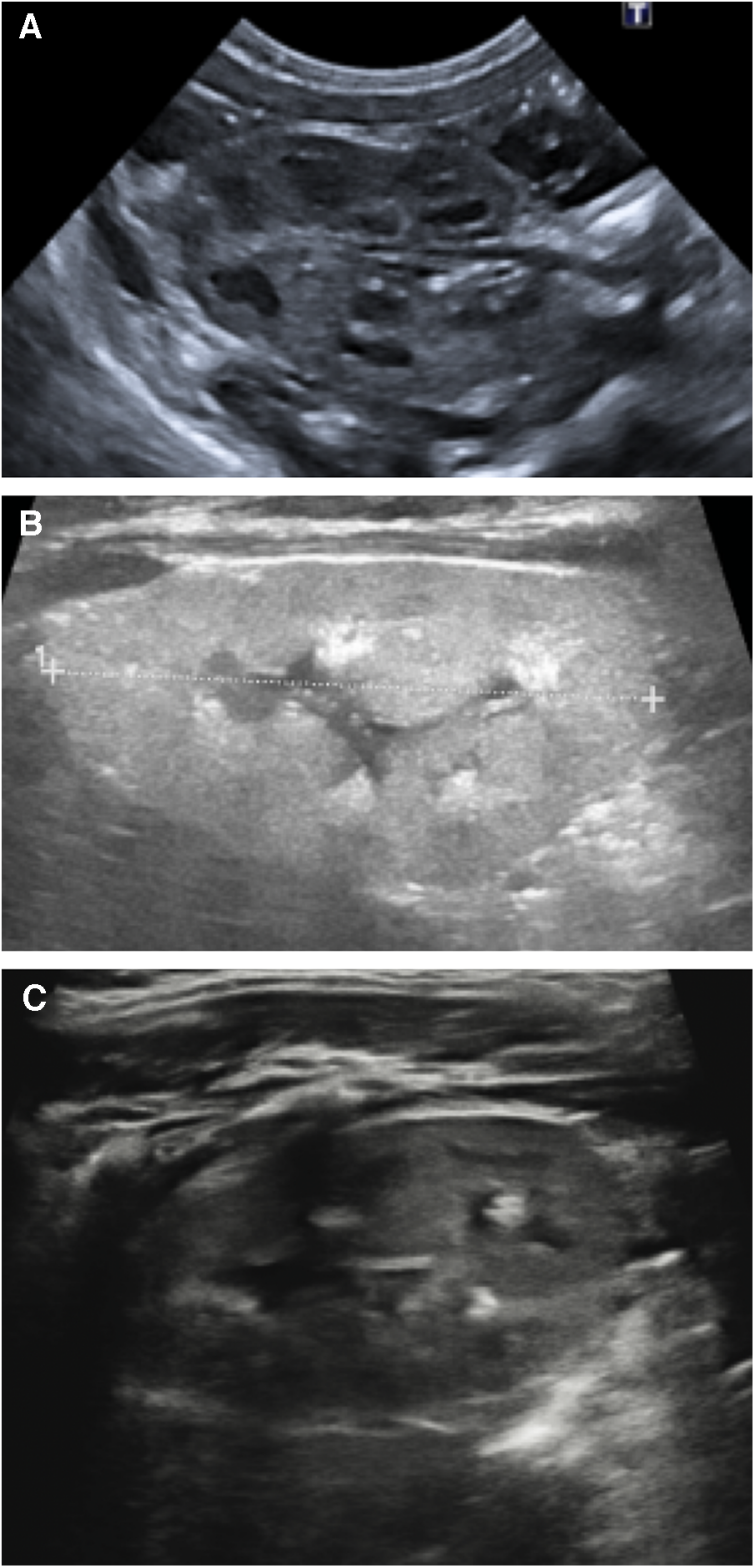
(**A**) Ultrasound scan at birth; (**B**) ultrasound scan at 3 months of age; and (**C**) ultrasound scan at 9 months of age.

The baby underwent serial ultrasound scans showing nephrocalcinosis at 3 months of age, and then, at 9 months of age, concomitantly with acute pyelonephritis, two hyperechogenic images with slight posterior acoustic shadowing of 6 and 5 mm, respectively, at the left lower renal pole, and a stone of about 6 mm in the premural tract of the right ureter, with modest dilation of the upstream ureter in the pelvic tract (5 mm) (Figure 1).

The patient was then placed on tamsulosin 0.2 mg daily with ureteral stone expulsion 3 days after starting the medication without symptoms. Tamsulosin therapy was recommended by our pediatric urologist team and prescribed off-label after parental consent. The stone composition was 60% calcium oxalate dihydrate and 40% calcium oxalate monohydrate.

**Figure 2:**
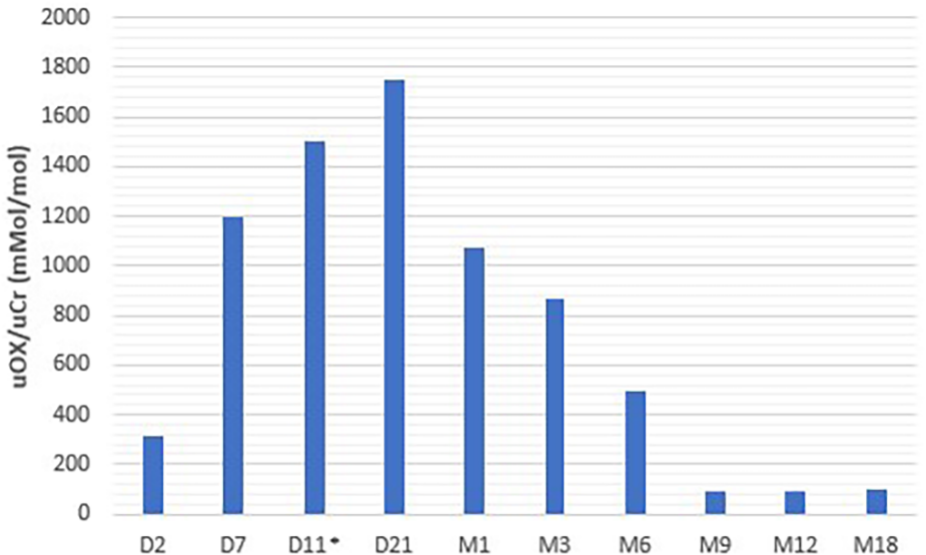
Urinary oxalate levels.

**Figure 3:**
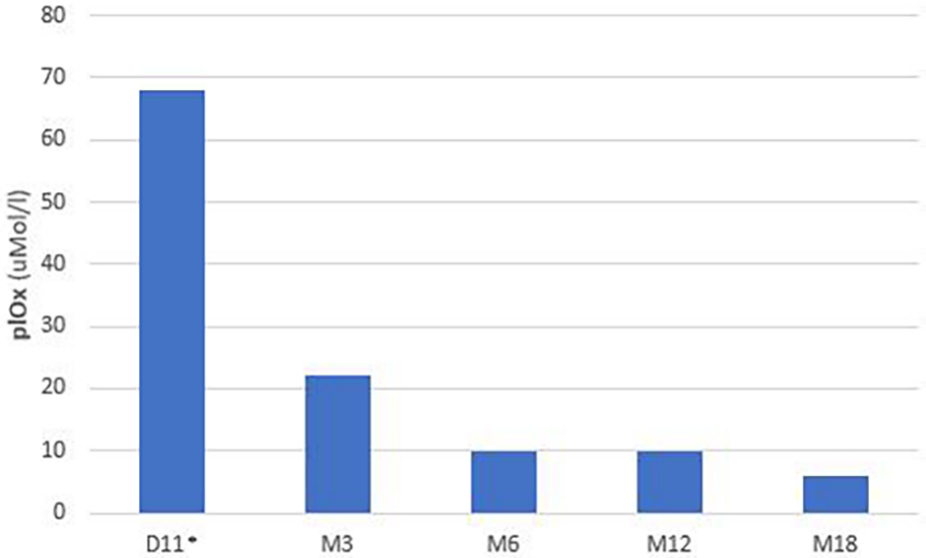
Plasma oxalate levels.

Urinary and plasma oxalate levels were monitored over time and are shown in Figures 2, 3, respectively.

At 12 and 20 months of age, the child showed growth according to the birth percentile.

At 20 months of age, the baby showed normal urinary oxalate values and kidney function, while the plasma oxalate level was 6 uMol/L. GFR matured normally during the first year of life and the infant presented a normal renal function from birth to the last follow-up. No signs of systemic oxalosis were observed.

The ultrasound scan at 12 and 20 months showed improvement in nephrocalcinosis and persistence of the two left kidney stones, unchanged from the previous examination. The baby showed no signs of systemic oxalosis at 20 months of age.

No adverse effect associated with the use of lumasiran was observed during the treatment.

## Discussion

The patient described in this case report is, to the best of our knowledge, the only patient born before 37 weeks of gestation who underwent treatment with lumasiran.

The efficacy of lumasiran in the pediatric cohort was demonstrated in the ILLUMINATE B trial (12), which enrolled 18 patients aged <6 years (range 3–72 months). Therefore, data on the use of this drug in newborns are currently limited, and systematic clinical studies could be useful to evaluate its effects and pharmacokinetics in the early stages of life. The experience regarding the use of lumasiran in the early stages of life was described by Mèaux et al. (13) who reported three cases of young patients with PH1. The first patient had a similar history to the patient reported here since she received a prenatal diagnosis based on family history and started treatment at 9 days of age, combining hyperhydration and potassium citrate. Again, the ultrasound examination, which was normal at birth, showed worsening around 2 months of age with the appearance of nephrocalcinosis. In this case, ultrasound worsening was related to persistently elevated urinary oxalate levels in the first 2 months of life, so the dose of lumasiran was increased to lower oxalate concentrations. At 10 months of age, nephrocalcinosis improved and kidney function was normal. In the other two cases described by Mèaux et al. (13), the patients started treatment at around 3 months of age, and both had nephrocalcinosis with normal kidney function. After 9 and 5 months of treatment, nephrocalcinosis improved.

The case described here has features very similar to those of the infants described by Mèaux et al. (13). In particular, the history and timing of diagnosis and initiation of treatment are similar to those of the infant described by them. In our case, it was possible to start treatment no earlier than the tenth day of life because approval for the use of lumasiran was sought from the Ethics Committee (lumasiran was not yet available in the market at the time, but available for compassionate use according to the Ministerial Decree September 7, 2017).

The uOxalate/uCreatinine ratio was used to evaluate urinary oxalate excretion in our patient, as well as in young patients described by other authors (13). Low GFR is associated with low urinary creatinine excretion in the first months of life, so urine oxalate/creatinine ratio could not be a reliable measure of oxalate excretion in early age. However, some evidence could support the use of the ratio also in infants (14).

A study conducted by Sonntag et al. on premature infants in the first weeks of life compared the oxalate/creatinine ratio with oxalate excretion in 24-h urine collection. The authors concluded that the oxalate/creatinine ratio in spot urine samples is suitable for screening for hyperoxaluria. Moreover in the ILLUMINATE B trial, the oxalate/creatinine ratio was used during the first year of life to evaluate urinary oxalate excretion.

Our data, in agreement with those of Mèaux et al. (13), demonstrate that the reduction in urinary oxalate is not immediate after treatment initiation, but has a window period of at least 15 days in which oxalate levels are persistently high, which may have resulted in oxalate deposition in the kidneys with subsequent development of nephrocalcinosis and urolithiasis.

Renal outcome is very difficult to predict in PH1. A specific genotype–phenotype correlation is difficult to obtain in patients with PH1 because environmental factors and modifier genes may play an important role in the clinical manifestations of the disease (15).

A retrospective study of 932 patients with PH1 included in the Oxal-Europe registry analyzed the impact of genotype, nephrocalcinosis, urolithiasis, and urinary oxalate excretion on renal function. Homozygosity for AGXT null variants and nephrocalcinosis were the strongest determinants for kidney failure in PH1 (16).

The case reported here presented early nephrocalcinosis, despite tempestive diagnosis and treatment. Nephrocalcinosis improved during the first year of life but a longer follow-up will be necessary to evaluate the medium-long-term effects of early therapy with lumasiran.

## Conclusion

To the best of our knowledge, the case reported here describes the first late preterm baby treated with lumasiran at birth, with a 20-month follow-up. The use of lumasiran appears to be safe in this type of patient. To date, early treatment of newborns with antenatal diagnosis of PH1 represents a therapeutic challenge for pediatric nephrologists.

## Data Availability

The raw data supporting the conclusions of this article will be made available by the authors, without undue reservation.

## References

[1] Hoppe B. An update on primary hyperoxaluria. Nat Rev Nephrol. (2012) 8(8):467–75. 10.1038/nrneph.2012.113 22688746

[2] Milliner DS Harris PC Sas DJ Cogal AG Lieske JC. Primary hyperoxaluria type 1. In: Adam MP Ardinger HH Pagon RA Wallace SE Bean LJH Stephens K, editors. Genereviews®. Seattle, WA: University of Washington (2002). p. 1993–2020. 20301460

[3] Oppici E Montioli R Cellini B. Liver peroxisomal alanine:glyoxylate aminotransferase and the effects of mutations associated with primary hyperoxaluria type I: an overview. Biochim Biophys Acta. (2015) 1854(9):1212–9. 10.1016/j.bbapap.2014.12.029 25620715

[4] Fargue S Acquaviva Bourdain C. Primary hyperoxaluria type 1: pathophysiology and genetics. Clin Kidney J. (2022) 15(Suppl 1):i4–8. 10.1093/ckj/sfab217 35592619 PMC9113437

[5] Cochat P Deloraine A Rotily M Olive F Liponski I Deries N. Epidemiology of primary hyperoxaluria type 1. Société de néphrologie and the société de néphrologie pédiatrique. Nephrol Dial Transplant. (1995) 10(Suppl 8):3–7. 10.1093/ndt/10.supp8.3 8592622

[6] Harambat J van Stralen KJ Espinosa L Groothoff JW Hulton SA Cerkauskiene R Characteristics and outcomes of children with primary oxalosis requiring renal replacement therapy. Clin J Am Soc Nephrol. (2012) 7(3):458–65. 10.2215/CJN.07430711 22223608 PMC3302673

[7] Cochat P Rumsby G. Primary hyperoxaluria. N Engl J Med. (2013) 369(7):649–58. 10.1056/NEJMra1301564 23944302

[8] Hoppe B Hesse A Brömme S Rietschel E Michalk D. Urinary excretion substances in patients with cystic fibrosis: risk of urolithiasis? Pediatr Nephrol. (1998) 12(4):275–9. 10.1007/s004670050452 9655356

[9] Groothoff JW Metry E Deesker L Garrelfs S Acquaviva C Almardini R Clinical practice recommendations for primary hyperoxaluria: an expert consensus statement from ERKNet and OxalEurope. Nat Rev Nephrol. (2023) 19(3):194–211. 10.1038/s41581-022-00661-1 36604599

[10] Cochat P Groothoff J. Primary hyperoxaluria type 1: practical and ethical issues. Pediatr Nephrol. (2013) 28(12):2273–81. 10.1007/s00467-013-2444-5 23494551

[11] Groothoff JW Metry E Deesker L Garrelfs S Acquaviva C Almardini R Lumasiran, an RNAi therapeutic for primary hyperoxaluria type 1. N Engl J Med. (2021) 384(13):1216–26. 10.1056/NEJMoa2021712 33789010

[12] Hayes W Sas DJ Magen D Shasha-Lavsky H Michael M Sellier-Leclerc AL Efficacy and safety of lumasiran for infants and young children with primary hyperoxaluria type 1: 12-month analysis of the phase 3 ILLUMINATE-B trial. Pediatr Nephrol. (2023) 38(4):1075–86. 10.1007/s00467-022-05684-1 35913563 PMC9925547

[13] Méaux MN Sellier-Leclerc AL Acquaviva-Bourdain C Harambat J Allard L Bacchetta J. The effect of lumasiran therapy for primary hyperoxaluria type 1 in small infants. Pediatr Nephrol. (2022) 37(4):907–11. 10.1007/s00467-021-05393-1 35015123

[14] Sonntag J Schaub J. The identification of hyperoxaluria in very low-birthweight infants–which urine sampling method? Pediatr Nephrol. (1997) 11(2):205–7. 10.1007/s004670050261 9090665

[15] Beck BB Hoppe B. Is there a genotype-phenotype correlation in primary hyperoxaluria type 1? Kidney Int. (2006) 70(6):984–6. 10.1038/sj.ki.5001797 16957746

[16] Metry EL Garrelfs SF Deesker LJ Acquaviva C D'Ambrosio V Bacchetta J Determinants of kidney failure in primary hyperoxaluria type 1: findings of the European hyperoxaluria consortium. Kidney Int Rep. (2023) 8(10):2029–42. 10.1016/j.ekir.2023.07.025 37849991 PMC10577369

